# Stack effects in tall building fires: a case study of Taiwan old apartment fire

**DOI:** 10.1038/s41598-022-13118-z

**Published:** 2022-05-27

**Authors:** HongSheng Huang, ChingYuan Lin, ShiuanCheng Wang, ChungHwei Su, LiPeng Chen

**Affiliations:** 1 Department of Architecture, National Taiwan University of Science and Technology, Taipei, 10607 Taiwan; 2 Department of Public Safety and Fire Science, Chia Nan University of Pharmacy and Science, Tainan City, 71710 Taiwan; 3 Department of Safety, Health and Environmental Engineering, National Kaohsiung University of Science and Technology, No. 1, Daxue Rd., Yanchao Dist., Kaohsiung City, 82445 Taiwan

**Keywords:** Engineering, Physics

## Abstract

Tainan, a city that prospered early in Taiwan, has a hot and humid atmosphere. Hence, the grilled doors in numerous old buildings for ventilation and lighting to conserve energy. This study analyzed a fire incident that occurred during the late night of March 17, 2019 in a 38-year-old dwelling, where three residents were severely covered with soot. The site investigation showed that eight staircases lead to the same basement, which apparently created a stack effect and a makeup air phenomenon. Numerical simulations have been performed in this study to reconstruct the fire scene, whose results were consistent with the actual fire scene. In particular, the results showed that some staircases in the fire were blackened by smoke, while others acted as makeup air inlets. The temperature at the households’ doors on all floors of Staircase 2, which was closest to the fire, exceeded 60 °C after four minutes. Furthermore, two immediately feasible improvement strategies according to the control volume theory of fluid mechanics were proposed in this study. Firstly, changing the grilled doors in the basement to a closed flat door style could effectively prevent smoke from flowing up in the staircases. Secondly, residents may consider closing the windows of the stairs at night to improve fire safety. The results showed that the chimney effect can be significantly reduced. These improvements could be a reference for other old dwellings to enhance their fire safety.

## Research motivation

### Taiwan’s ancient capital: Tainan

Located in the southwest of Taiwan, Tainan City experiences hot and humid weather, especially during summers, with an annual average temperature between 26 and 33 °C^1^. It is the earliest developed city in Taiwan and holds a history of 350 years. The urban development histories of other famous cities, such as Taipei City and Kaohsiung City, are shorter than Tainan City. Hence, there are numerous old buildings in Tainan^2–4^.

In 1996, Taiwan’s fire regulations were greatly improved^5^. The fire equipment of the old buildings constructed before 1996 only met the minimum safety criteria. Some of the old buildings were only equipped with fire extinguishers and lacked other fire safety equipment. In group dwelling fires, residents could only be evacuated to the ground through internal staircases. Therefore, some residents would purchase additional refuge and escape equipment, such as emergency ladders, rescue cords, sliding rods, or escape slings^6,7^.

### Smoke hazard in dwelling fires

In Taiwan, dwelling fires are the most common among fire incidents. As shown in Table 1, the fire incidents in the last three years in standalone and group dwellings account for more than 75% of all fires^8^. Fire safety in apartment fires is one of the important issues in every country. Liu and Chow used the temperature of the hot smoke layer as a system state variable to analyze the occurrence of flashover based on a two-zone model^9^. Lin et al. used Fire Dynamics Simulator (FDS) to simulate a fire accident in an old five-story apartment building in New Taipei City, Taiwan. The calculated temperature and smoke field distribution at the fire site were consistent with the reports of the fire department^10^. Ghassempour et al. found that fires in socio-economically disadvantaged areas among males and adults were often overlooked and not reported^11^. Jonsson et al found that fatal residential fires more often originated in the bedroom, were more likely to occur at night, and were more often caused by smoking in Sweden^12^. The situation is very similar to Taiwan^13^.

**Table 1:** Fire case statistics in the last 3 years in Taiwan.

Year		Standalone dwelling	Group dwelling	Office building	Business building	Compound building	Factory	Warehouse	Temple	Others
2017	Fire number	3365	3651	122	336	102	294	719	66	439
	Percentage (%)	37.0	40.1	1.3	3.7	1.1	3.2	7.9	0.7	4.8
2018	Fire number	3057	3614	119	265	118	341	690	53	508
	Percentage (%)	34.9	41.2	1.4	3.0	1.3	3.9	7.9	0.6	5.8
2019	Fire number	2900	3266	120	250	94	357	569	49	398
	Percentage (%)	36.2	40.8	1.5	3.1	1.2	4.5	7.1	0.6	5.0

**Figure 1:**
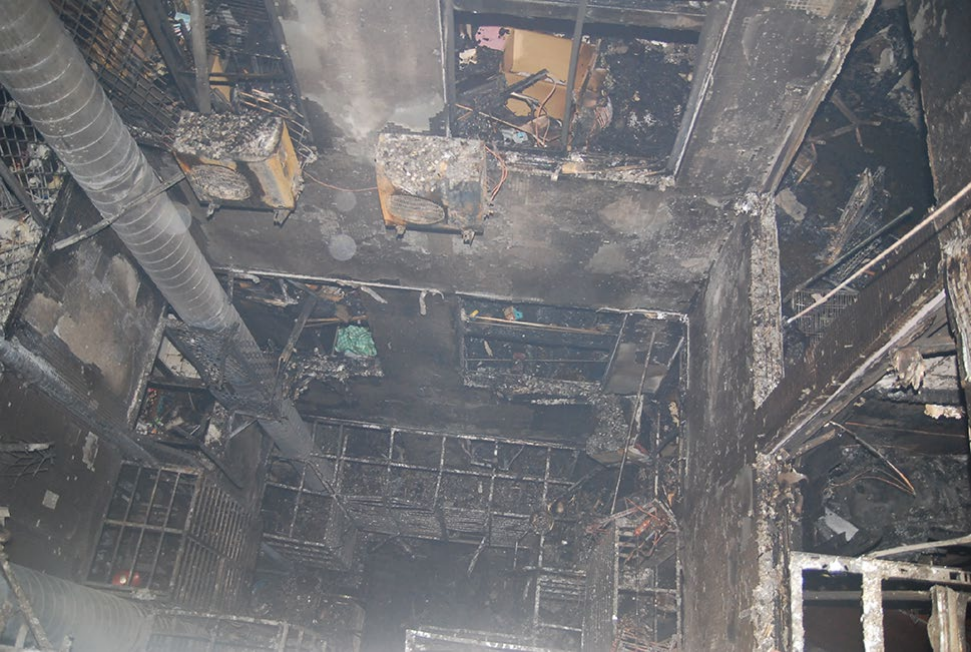
Burned situation in the atrium of the high-rise residential fire.

The stack effect commonly occurs during fires in high-rise buildings. Figure 1 shows a fire burning upward through a shaft in a fire case. Ferreira and Cutonilli found that the stack effect is common in multi-story buildings, especially in high-rise ones^14^.

**Figure 2:**
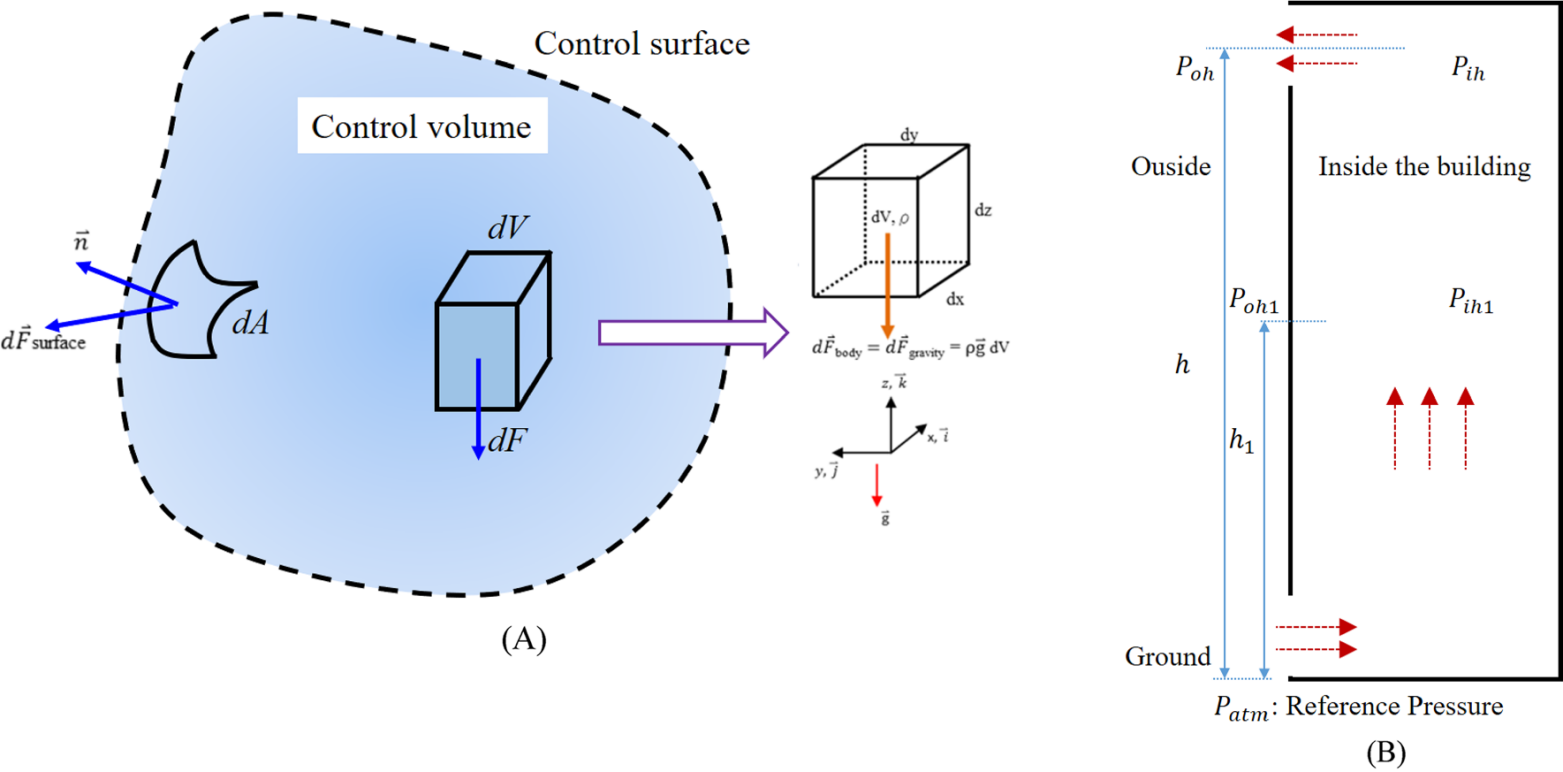
The control volume theory of fluid mechanics (**A**) Force distribution on the fluid. (**B**) Stack effect in a tall building fire.

All fluid stresses are as shown in Fig. 2A and expressed as Eq. (1)^15^, according to the control volume theory of fluid mechanics. Usually, air density is considered a constant value because there is no significant difference between a building’s inside and outside temperatures. Therefore, the buoyancy of the indoor air is not significant. When a building is on fire, the temperature at the fire site will rise sharply by hundreds of degrees Celsius. At the same time, when the density of heated air drops sharply, the buoyancy phenomenon becomes obvious. Furthermore, the stack effect occurs in a vertical space.

1 $$\sum \overrightarrow{F}=\sum {\overrightarrow{F}}_{body}+\sum {\overrightarrow{F}}_{surface}=\sum {\overrightarrow{F}}_{body}+\sum \left({\overrightarrow{F}}_{surface}+{\overrightarrow{F}}_{viscous}\right)={\int }_{CV}\rho \overrightarrow{g}dV+{\int }_{CS}{\sigma }_{ij}\cdot \overrightarrow{n}dA$$

Herein, $$F{:}\,force(N),\,{\sigma }_{ij}{:}\,fluid\,stress\,in\,x,y,z\,direction, \rho{:}\,density(\mathrm{kg}/{\mathrm{m}}^{3}), \overrightarrow{n}=normal\,vector, g{:}\,gravity\,vector\left(\mathrm{m}/{\mathrm{s}}^{2}\right)$$.

It causes the fire to burn more vigorously due to the makeup air phenomenon. Figure 2B shows the stack effect at the fire site^16,17^.

The vertical pressure distribution in the shaft is estimated by equations derived from hydrostatic models, such as Eq. (2)^18–20^. The pressure difference Δ*P*_*h*_ between pressures inside and outside at height h of the shaft is:

2 $${\Delta P}_{h}={P}_{ih}-{P}_{oh}$$

Through the gas constant *R* and the atmospheric pressure *P*_*atm*_, Eq. (2) can be expressed as Eq. (3) in terms of the reference pressure difference between the shaft’s ΔP_h1_ inside and outside at height h_1_:

3 $${\Delta P}_{h}={\Delta P}_{h1}+\frac{{P}_{atm}\cdot g\left({h-h}_{1}\right)}{R}\left(\frac{1}{{T}_{o}}-\frac{1}{{T}_{i}}\right)$$

The second term *P*_*atm*_ g/*R* is simplified as *Ks* of 3460 NK m^−3^. Equation (3) is derived as follows:

4 $${\Delta P}_{h}={\Delta P}_{h1}+{K}_{s}\left(\frac{1}{{T}_{o}}-\frac{1}{{T}_{i}}\right)h-{K}_{s}\left(\frac{1}{{T}_{o}}-\frac{1}{{T}_{i}}\right){h}_{1}$$

Herein, *h*: height (m), ${P}_{ih} , {P}_{oh}$: pressures inside and outside at height h (Pa), ${T}_{i} , {T}_{o}$: inside and outside temperature (K), *R*: gas constant (m^3^ Pa K^−1^ mol^−1^), *P*_*atm*_: atmospheric pressure (Pa), *Ks*: 3460 NK m^−3^, ΔP_h1_: pressure difference between inside and outside of the shaft at height *h*_*1*_ (Pa).

This study analyzed a dwelling fire that happened in Tainan City at 00:04 on March 17, 2019. The building was a 38-year-old dwelling. Although there were no casualties, more than 50 households in the building were still affected because the fire broke out late at night. The fire officials regarded it as a major fire, wherein 26 fire engines and 65 firefighters were dispatched to the fire scene. Many similar buildings in Tainan City are only equipped with fire extinguishers and lack other fire safety equipment, such as fire detectors or alarms. Thus, based on a fire scene reconstruction, this study proposed measures for immediate and inexpensive improvements that could be used as a reference for similar old dwellings to enhance their fire safety.

## Fire case description

The case happened at 00:04 a.m. in the basement, mainly used as a parking garage. The two driveway entrances were located near Staircase 3 and Staircase 6, respectively. With an entrance width of 2.0 m, it was designed for motorcycles only. There were a total of eight staircases in the building, all connected to the same basement. The motorcycles on fire were near the door of Staircase 2, three of which were burnt. The detailed records of the accident are shown in Table 2.

**Table 2:** The fire situation of the analyzed case.

Fire scene	Situation
Time on fire	00:04 a.m
Fire was extinguished	00:32 a.m
Fire location	Basement B1 of the second 2nd stair
Cause of fire	3 locomotives caught fire burning area is about 2.6 m^2^
Evacuated households	3 buildings with 54 households in total
Number of injured	15 were sent to hospital, 0 death
State of the occupants in the fire	Many people egressed by themselves
	39 people trapped and rescued by firefighters

**Figure 3:**
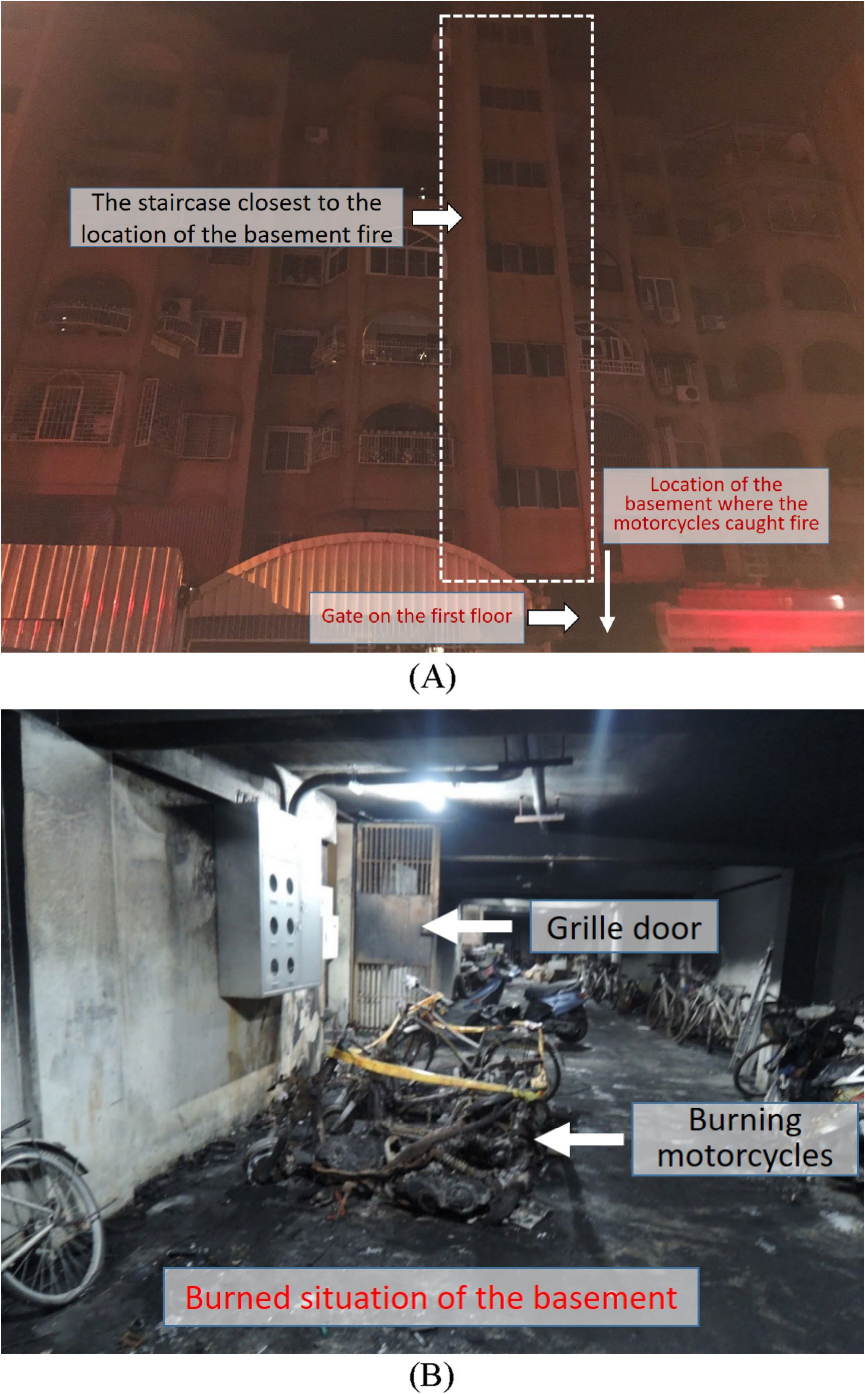
Description of the analyzed case (**A**) The appearance of the building. (**B**) The fire location in the basement.

**Figure 4:**
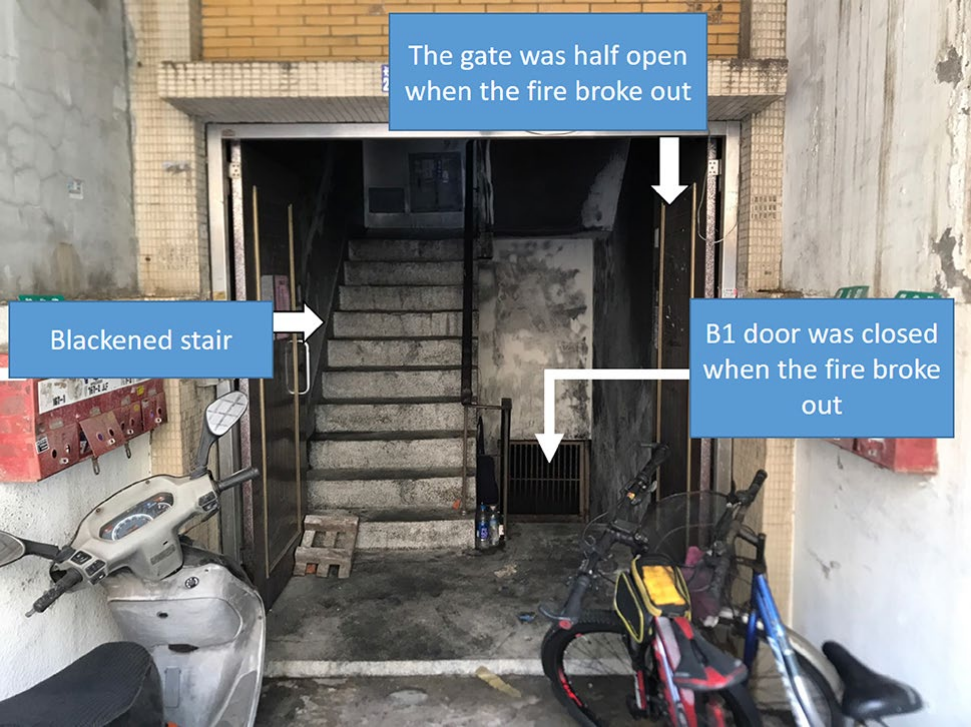
Blackened gate on the first floor.

**Figure 5:**
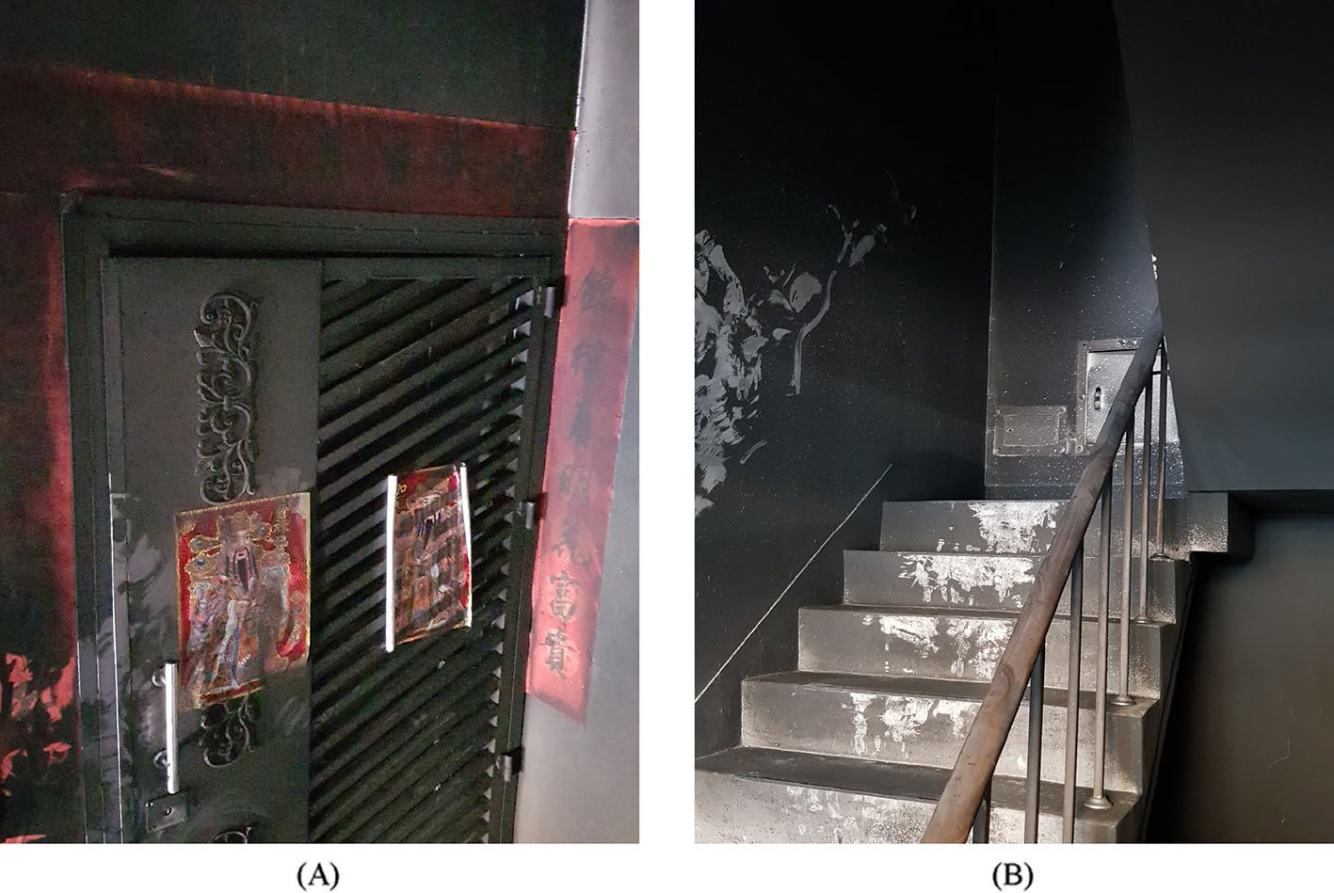
Description of the blackened stair (**A**) Doorway of residents on the third floor. (**B**) Steps of the stair on the third floor.

Due to the lack of a fire detector or alarm system, the residents on the higher floors failed to discover the fire early. At the time of evacuation, the fire was already severe, making it difficult to rescue the residents from the site. The mouths and noses of evacuated residents were blackened due to a large amount of smoke. The fire scene is shown in Fig. 3A. On the other hand, Fig. 3B shows the scene in the basement. The cause of the fire was “suspected arson”. The hot smoke flowed upward along the staircases, as seen in Figs. 4 and 5, which show the blackened inside of Staircase 2.

**Figure 6:**
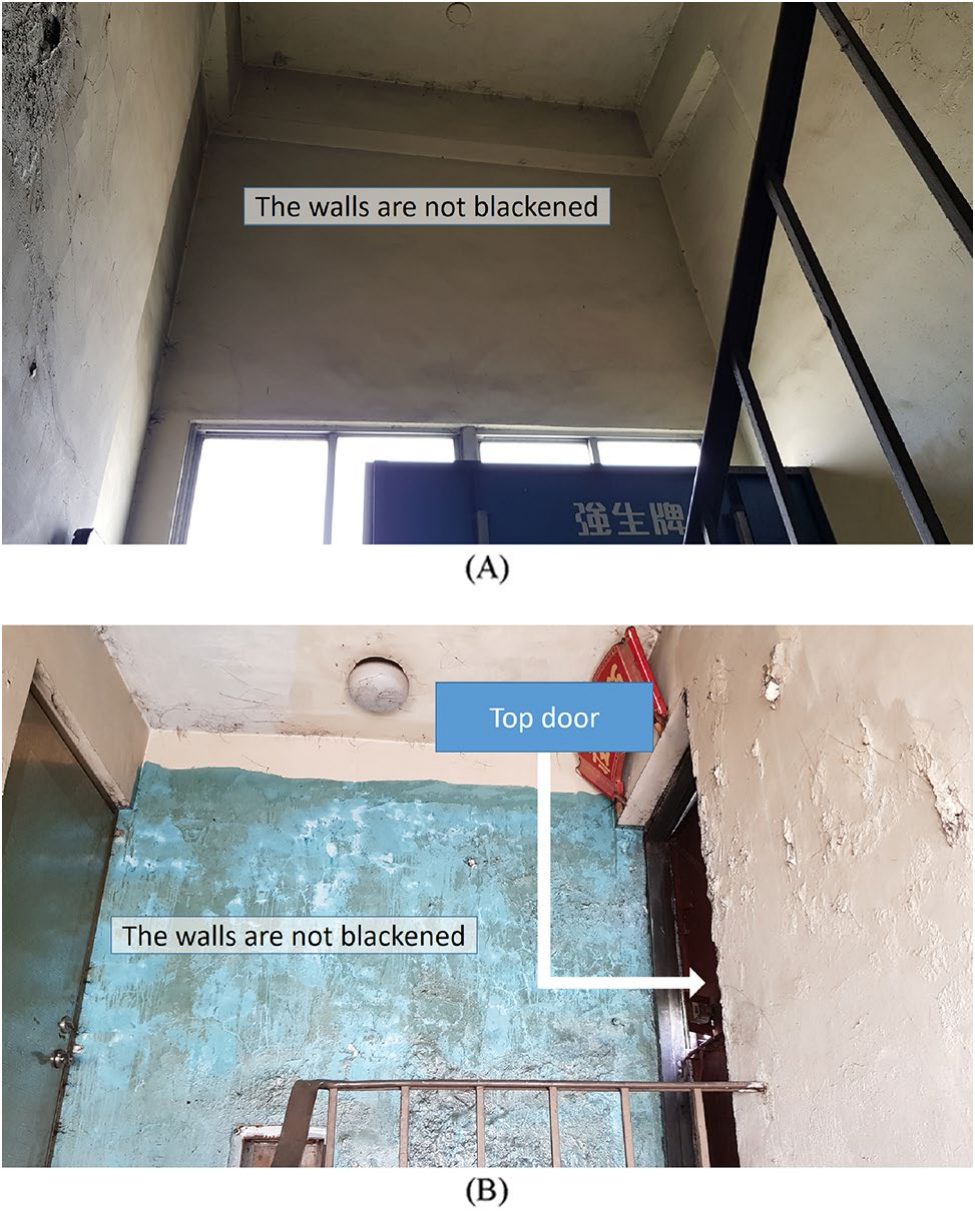
Description of the highest position of the fire (**A**) The highest platform of the stair was not blackened by hot smoke. (**B**) The highest floor of the stair was not blackened by hot smoke.

Interestingly, in this case, the area at the top of Staircase 2 was not blackened. In particular, Figs. 6A and B show that the walls and floors were intact and not blackened. Further, the site observation found that two windows at the top of Staircase 2 were closed.

## Research methodology

### Numerical analysis method

Computer functions and numerical calculation abilities have been greatly improved with the advancement of computer technology. FDS, developed by the Building and Fire Research Laboratory of the National Institute of Standards and Technology (NIST), can be used to reconstruct complex fire scenes, regardless if it is a building, factory, or tunnel fire. The grid’s temperature, velocity, pressure, visibility, and concentration can be calculated accurately using the governing equations^21^.

Musser and McGrattan used FDS to simulate four smoke flows in a room: forced convection, natural convection, mixed convection, and displacement ventilation^22^. Lin et al. utilized two types of fire simulation software—CFAST and FDS—to reconstruct a motorcycle shop fire accident in Taiwan^23^.

FDS is based on large eddy simulation (LES) to simulate 3D buoyancy-driven airflow at a fire scene. The software framework consists of a numerical calculation engine, pre-processing, and post-processing stages^24^. With technological development, the field model has developed into an effective model that can reduce the calculation time for studying fire science.

The grid sizes obtained by numerical computation can directly affect the simulation results. The model grid setting must consider the computing speed and correctness—the primary considerations in the numerical simulation process^25,26^. The characteristic fire diameter was used in this study to evaluate and analyze the optimal grid size at a specific heat release rate. McCaffery indicated that the minimum length scale of a fire plume is the characteristic fire diameter, D*, which can be used to determine the grid size^27^ and is expressed as Eq. (5):

5 $${D}^{*}=[\frac{\dot{Q}}{{\rho }_{\infty }{C}_{\infty }{T}_{\infty }\sqrt{g}}{]}^2/5$$

where $\dot{Q}$: total heat release rate (kW), ${T}_{\infty }$: space temperature (K), ${\rho }_{\infty }$: air density (kg/m^3^), g: gravitational acceleration (m/s^2^), ${C}_{\infty }$: air specific heat (kJ/kg K).

Ji et al.^28^ and McGrattan et al.^29^ pointed out that setting the grid size to 0.1 times the characteristic flame length can reach good efficiency and accuracy based on the balance between the experimental conditions and the computer operation time. Although a few methods are suggested to determine the grid sizes for a fire simulation, there is still a need to carry out sensitivity analysis and perform few numerical tests^30^.

### Simulation model and parameter settings

Smoke is the leading cause of death in a fire. In addition to reducing visibility at the fire site, smoke also affects evacuation decisions and delays evacuation. Table 3 shows Taiwan’s regulations on the tolerable environment for people at a fire site^31,32^. This study referred to Lin^33,34^ and Chang^35^ for the heat release rate of motorcycle burning. The actual motorcycle burning was conducted in this study and was measured using a cone calorimeter apparatus. The results showed that when three motorcycles burnt and the fire grew according to the T-square theory, the growth coefficient would be 0.00019 MW/s^2^. The average heat release rate during the peak period of motorcycle combustion is between 0.967 and 1.202 MW. When modeling the combustion behavior of motorcycles, the maximum heat release rate is set as 1.106 MW. Measurements of the burning of two and three motorcycles found that the maximum heat release rates were 2.51 and 5.44 MW, respectively^34^.

**Table 3:** Criteria for the safe environment of personnel in a fire scene in Taiwan.

Hazard type	Limit value
Temperature	Below 60 °C
CO concentration	Less than 1500 ppm
Visibility	Above 10 m

In 2000, MeHgret and Vauquelin developed a semi-empirical model to determine the physical characteristics of fire in tunnels. The combustion substance used was heptane^36^. In 2017, Chang et al. conducted two full-scale tunnel fire experiments using heptane as fuel^37^. Table 4 shows the simulation setting of this study. Heptane was set as the fire source in this study. Heptane is commonly used as a fuel for fire testing in road tunnels either in full-scale test activities^38^ or fire simulations to test ventilation systems and train fire brigades before each new tunnel opens^39^.

**Table 4:** Setting of parameters in numerical simulation.

Parameter	Value
Max heat release rate	5.44 MW
Size of the burning object	1.6 m(W) × 1.8 m(L) × 0.8 m(H)
Burning surface	5 (The upper surface and surroundings of the object)
Coefficient of fire growth	0.00019 MW/s^2^
Time to full growth	169.21 s
Fire source	Heptane
Size of platform window	0.6 m(W) × 1.0 m(H) × 2 m(windows)

**Figure 7:**
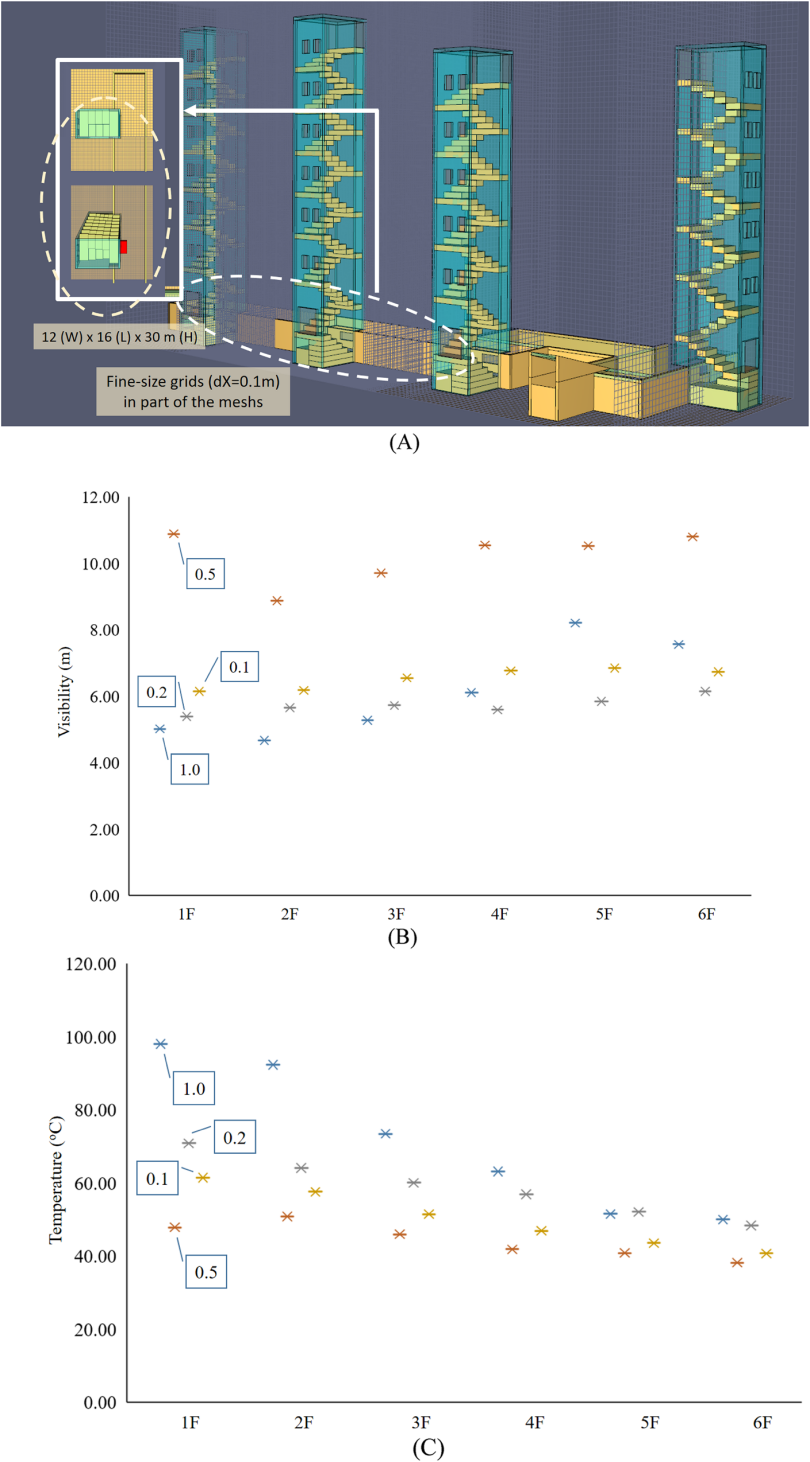
Sensitivity analysis of the tested model in four grid sizes (dx = 1.0, 0.5, 0.2, 0.1 mm). (**A**) Tested model. (**B**) Visibility. (**C**) Temperature.

By calculation, the 0.1D* value was 0.188 m. The model of each case has adopted the same grid size, and the computer hardware used is Intel i7-6700 with 16 GB RAM. The execution time for each case is approximately 71 h. There were approximately nine million grids. Notably, the number varied slightly in different cases. Figure 7 shows the results of this study’s grid size sensitivity analysis. The grid size near the fire source was 0.2 m. The spaces of the tested models were half of the original model, as shown in Fig. 7A. The settings of the fire sources were the same as the original model in this study. According to the results, these two parameters tend to stabilize after 300 s. The grid size of a part of the model (12 × 16 × 30 m) is set to 0.1 m, including the 2nd staircase, as shown in Fig. 7(A). Figure 7B and C are the sensitivity analysis of the grid sizes when the simulation is stable. The detection points on each floor were set on the platform of the stairs. The results illustrate that the visibility and temperature of each floor are oscillating convergence. All grid sizes are set to 0.2 m.

## Results and discussion

Firstly, the actual conditions of the fire scene in this study were scientifically reconstructed, which revealed a number of details during the discussion of the phenomena. Then, the stack effect was subsequently discussed, and the effectiveness of improvement measures was proposed for analysis. Table 5 shows the symbol descriptions of all cases. Case Tc_Wtc_B1o is the case at the time of fire and would be the case being compared. Some enhancement measures were explored in this study. The underlined text in Table 5 indicates that the three parameters have been changed because the main factors are the states of the air inlet and exhaust openings in Stair #2. A total of four cases were analyzed.

**Table 5:** Symbolic description of the simulation cases.

Case No	Openings in Stair #S2		Basement door of Stair #S2
	Top door*	Platform window/status	
Tc_Wtc_B1o	Close	Window Wt**/Close	Open
Tc_Wtc_B1c	Close	Window Wt/Close	Close
Tc_WAc_B1o	Close	All floors/Close	Open
To_WAo_B1o	Open	All floors/Open	Open

*Top door of the 2nd stair.

**Figure 8:**
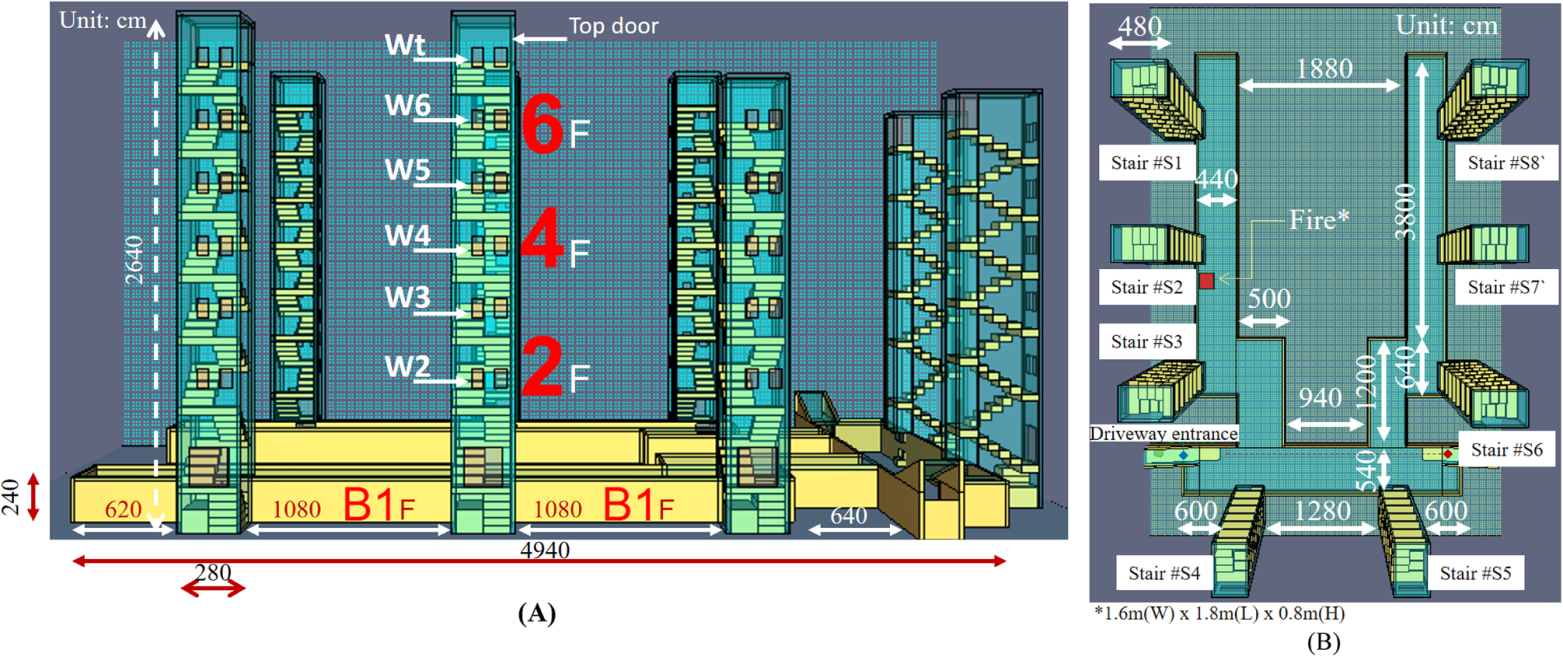
Configuration of the simulation model (**A**) Front view. (**B**) Top view.

**Platform window number is shown in Fig. 8A.

### Fire scene construction and analysis

In this study, FDS 6.6.0, the internationally famous fire simulation software, was used for simulation. Figures 8A and B show the established model. The building was built in 1982. There was no control door at the vehicle entrances, and the basement was usually open. In addition, there was no security guard in the basement, and the site investigation showed that security equipment, such as monitors, had not been installed.

Figure 8A shows the front view of the model, indicating eight staircases. The staircases were all connected to the same basement, with the dimensions shown in the figure. The two driveway entrances were located near Staircase 3 and Staircase 6, respectively. The motorcycle that caught fire was parked near the entrance to Staircase 2 in the basement, as shown in Fig. 8B.

**Figure 9:**
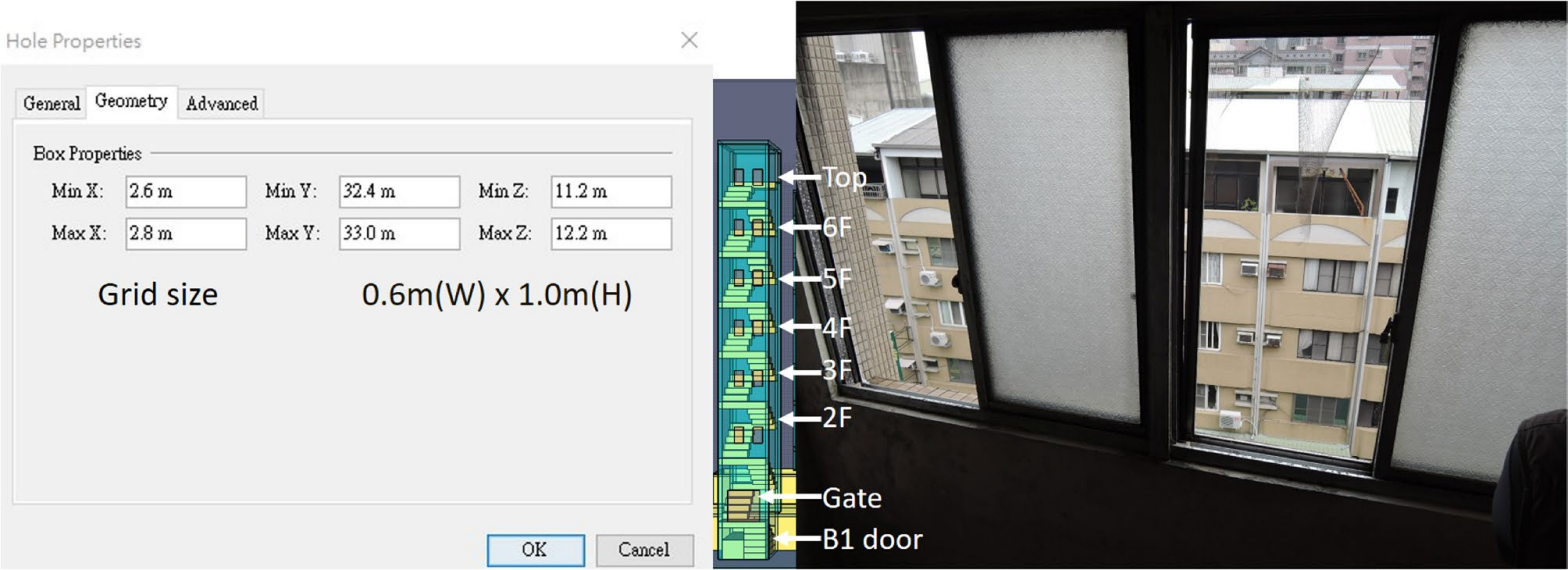
Setting value of parameters at the platform window of No. 2 staircase.

Furthermore, the staircases had landings. Each landing was equipped with two double-opened windows that provided ventilation and lighting. The residents indicated that most windows were kept open. Figure 9 shows the window appearance and the set values of the software. The windows were open, as shown in the figure. This study assumed that the glass would not break in a high-temperature environment.

**Figure 10:**
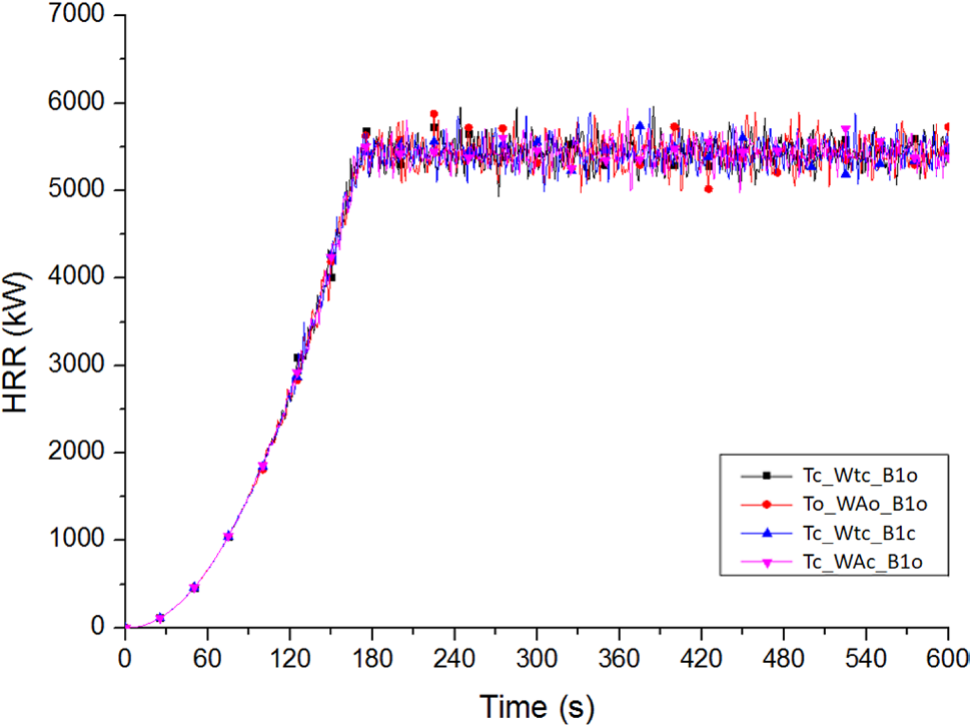
Heat release rate of each case.

Figure 10 shows the heat release rates (HRRs) in all cases. According to the report of Chen and Lin, the fire reached 5.44 MW at 169.21 s and continued to burn to 600 s^34^. The burning at a fire site will vary if doors and windows at different locations are open. However, considering that only one set of experimental data could be referred to, this study assumed that the HRRs in all cases were the same. This assumption also had the advantage of determining the effectiveness of various improvement measures under the same burning conditions.

#### Temperature distribution

**Figure 11:**
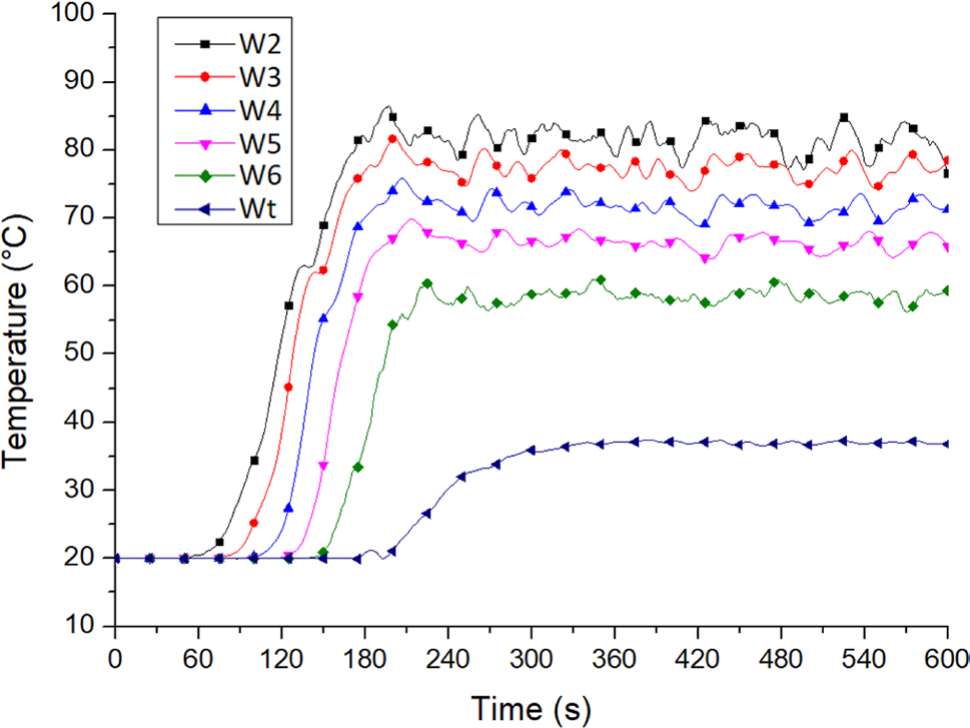
Temperature variation at the platform windows on each floor of No. 2 staircase (for Case Tc_Wtc_B1o).

Case Tc_Wtc_B1o is the simulation case of the fire site. Figure 11 shows the outflow temperature of the smoke from the windows on all floors of Staircase 2 after the motorcycles caught fire. The recording area was located in the middle of the window, 0.2 m away from the glass inside the building. The outflow temperature of the hot smoke from W2 (the landing window of the second floor) was the highest at 85 °C, followed by 80 °C at W3 (the window on the third floor). The temperature decreased in the higher floors, indicating the existence of a rising fire plume—a stack effect.

In Case Tc_Wtc_B1o, window Wt at the top was not open, and the measured temperature was only 36.5 °C. The temperature measured at window W6 was close to 60 °C, indicating that the hot air discharged from the window on the sixth floor had not flowed to the top of the staircase and that this location was not affected by the stack effect.

**Figure 12:**
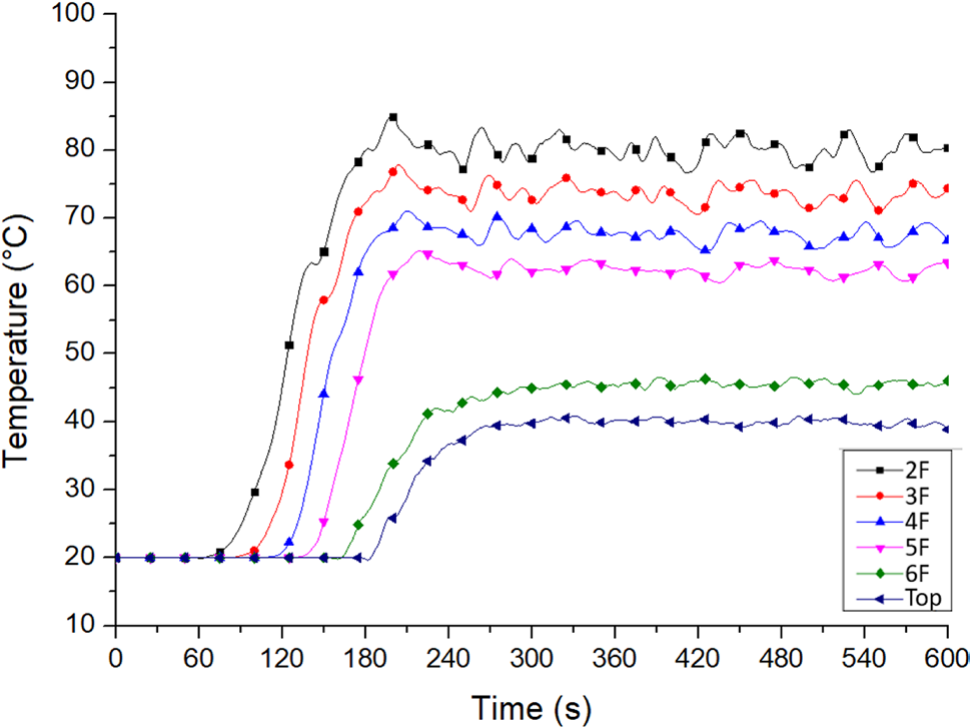
Temperature variation at the resident doors on each floor of No. 2 staircase (for Case Tc_Wtc_B1o).

Figure 12 shows the temperature changes at the residents’ doors on each floor near Staircase 2. Table 6 shows the maximum door temperature. This study referred to some research works of Klote et al., such as the Handbook of Smoke Control Engineering and the SFPE Handbook of Fire Protection Engineering. This phenomenon illustrates the influence of the stack effect in the shaft^40,41^. The simulation results showed that four minutes after the fire began, the temperature at all residents’ doors from the second to the fourth floor exceeded the acceptable temperature for the human body, namely 60 °C, as shown in Table 3. Therefore, it was not suitable to open the staircase doors to escape. The temperatures of the sixth floor and that at the top area were 46.2 and 40.3 °C, respectively, indicating that the hot air had not reached the two places and also meant that this location is not affected by the chimney effect.

**Table 6:** The highest temperature at the door of the residents on each floor of No. 2 staircase.

	Highest temperature (^o^C)			
	Tc_Wtc_B1o	To_WAo_B1o	Tc_Wtc_B1c	Tc_WAc_B1o
Top	40.3	53.0	20.0	20.0
6F	46.2	58.4	20.0	21.7
5F	64.8*	**62.8**	20.0	25.2
4F	**70.7**	**68.7**	20.0	31.5
3F	**78.0**	**76.5**	20.0	43.1
2F	**84.8**	**82.4**	20.0	**64.8**

*Bold value indicate that the value exceed the tolerable temperature of the human (60 °C).

#### Flowing hot air from windows

**Figure 13:**
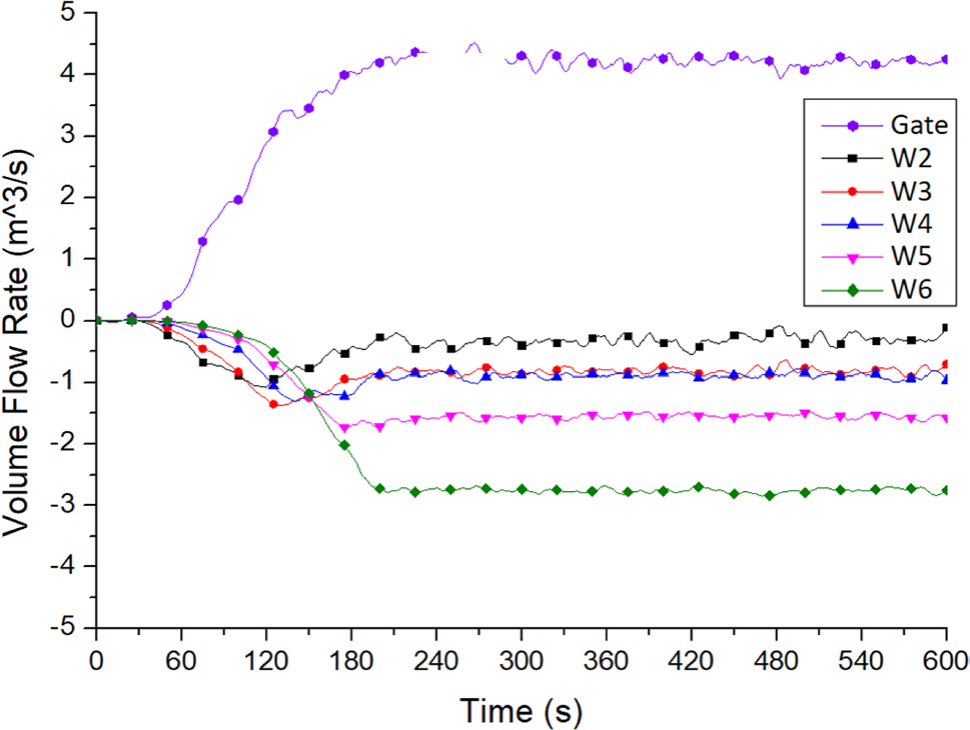
Flowrate variation at the platform windows on each floor of No. 2 staircase (for Case Tc_Wtc_B1o).

Figure 13 shows the hot air flow rates of the front entrance door and the windows on all floors. Positive values indicate the air flowing into the staircases, while negative values indicate the air flowing out of the staircases. The results of Case Tc_Wtc_B1o showed that window W6 had the highest hot air outflow rate of 2.9 m^3^/s, followed by window W5, which had a flow rate of 1.6 m^3^/s. The temperature of the hot air outflow from the window on the second floor was the highest. However, the outflow rate was only 0.5 m^3^/s, indicating that the hot air mainly flowed upward and not outward.

The curve value again proved the existence of a rising fire plume in the staircase, showing an obvious stack effect. The outside air from the front entrance door on the first floor flowed in at a rate of 4.2 m^3^/s, which could be regarded as the makeup air phenomenon. The flow of airflow showed the phenomenon of the typical stack effect.

#### Smoke diffusion

**Figure 14:**
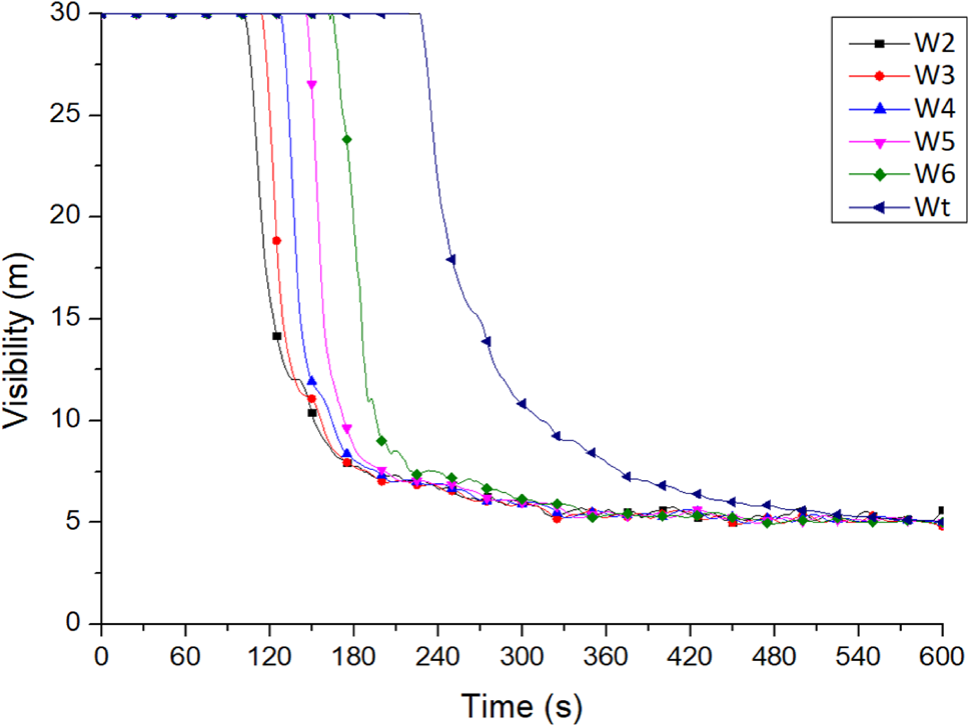
Visibility variation at the platform windows on each floor of No. 2 staircase (for Case Tc_Wtc_B1o).

The smoke from all windows was analyzed for its concentration, as shown in Fig. 14. The results showed that window W2 on the second floor discharged the highest concentration of smoke. At 151 s, the smoke concentration created visibility of less than 10 m. At the same time, as the floors rose, the smoke visibility gradually increased, indicating low smoke concentration. In addition, the smoke from window Wt on the top floor became dense when the fire had already been burning for some time. The visibility was attenuated to less than 10 m at about 313 s. The changes in the smoke concentration proved that the smoke gradually flowed upward because of the stack effect.

**Figure 15:**
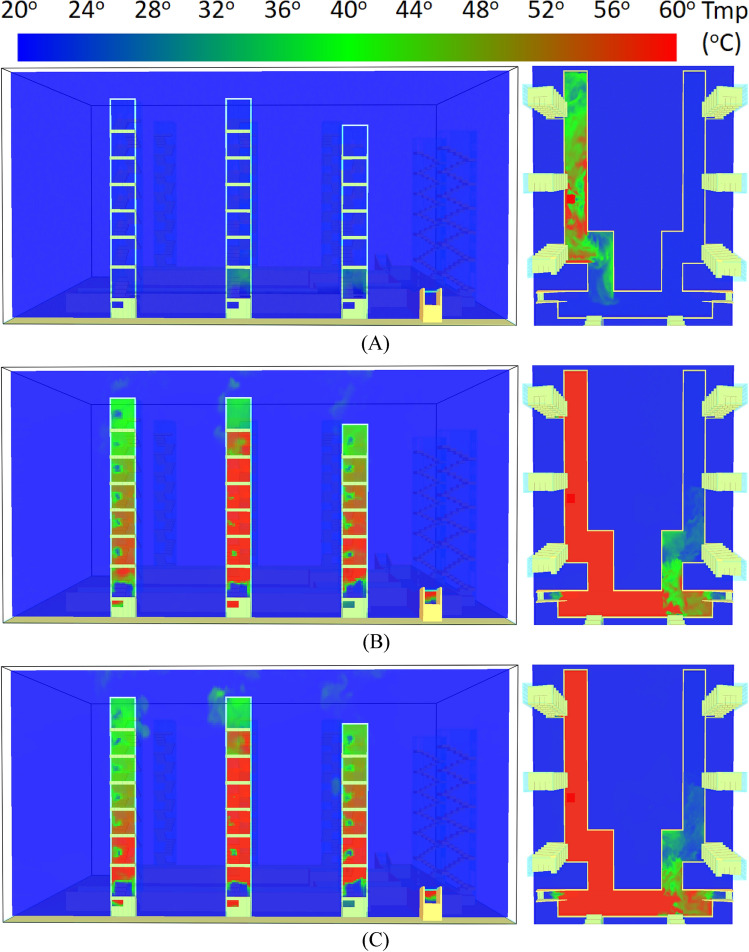
Smoke spread inside the stairs of buildings for Case Tc_Wtc_B1o (**A**) 60 s. (**B**) 300 s. (**C**) 600 s.

Figure 15 shows the hot air distribution of the entire building at 60, 300, and 600 s in Case Tc_Wtc_B1o. It was assumed in the simulation that the residents’ doors were all closed, and only the windows in the staircases were open. This study also assumed that window Wt of Staircase 2 was closed, while the windows on other floors were open. The results showed a noticeable stack effect and makeup air phenomenon because the staircases were connected to the same basement. At 60 s, as shown in Fig. 15A, the hot air rose in Staircases 1, 2, and 3. Figure 15B shows the temperature distribution at 300 s after the fire, indicating that the hot air rose. There was apparent high-temperature distribution in Staircase 2 from the basement to window W6. However, window Wt at the top had a low temperature.

On the other hand, the temperature of Staircases 6, 7, and 8 remained at 20 °C. It was suspected that the temperature was the inflow temperature of external air. This study concluded that the hot air failed to rise to the top because window Wt was closed. In the basement, the temperature from Staircase 1 to Staircase 5 was higher than 60 °C. Meanwhile, the temperature in Staircases 6, 7, and 8 remained at 20 °C, indicating that the three staircases were the inlets of the makeup air at the fire site.

The temperature distribution at 600 s after the fire is shown in Fig. 15C. The temperature distribution trend was similar to that shown in Fig. 15B, indicating a stable hot airflow. The figure also shows the low temperature of the two driveway entrances. It was suspected that these two places were the external air inlets.

**Figure 16:**
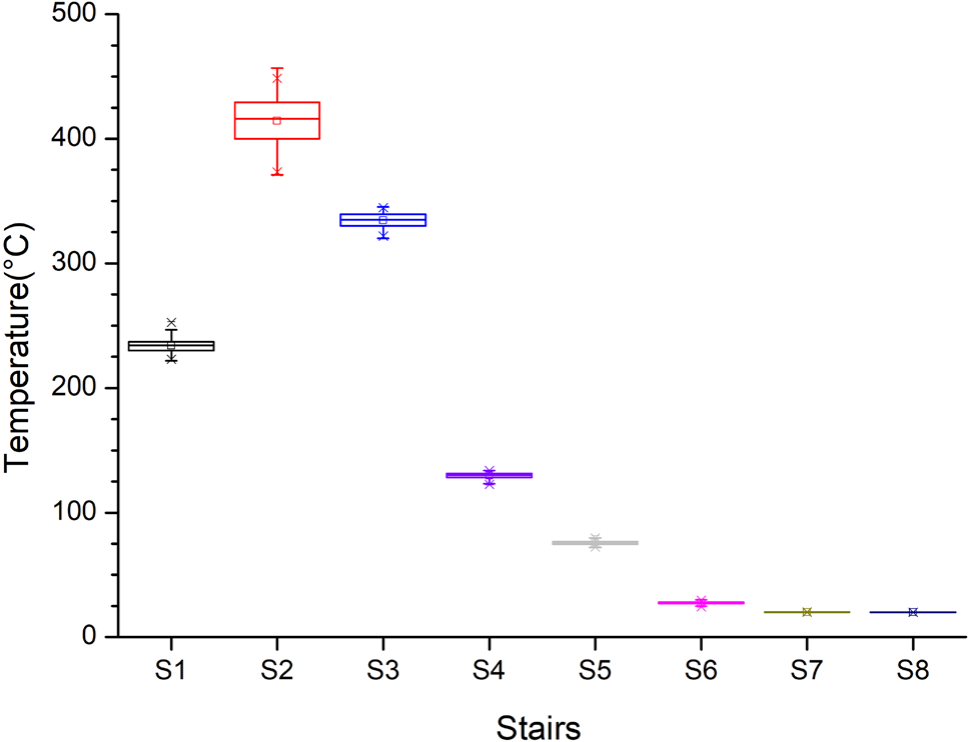
Temperature variation of B1 entrances at different stairs (for Case Tc_Wtc_B1o).

Figure 16 shows the temperature box plot of the entrances to the eight staircases in the basement. Figures 11 and 12 show the temperature from 240 to 600 s after burning was initiated and indicate the high temperature of Staircase 2 and the low temperatures of Staircases 6, 7, and 8. The figures demonstrate the phenomenon of external air inflow and that the hot air could not be diffused to the three staircases.

All the above phenomena were verified to be consistent with the actual conditions of the fire scene. The simulation results showed the apparent stack effect of all staircases and could explain why the top of Staircase 2 at the fire site was not affected by smoke and the walls not blackened. Therefore, the above analysis could provide a reference for developing improvement measures.

### Stack effect in staircases

**Figure 17:**
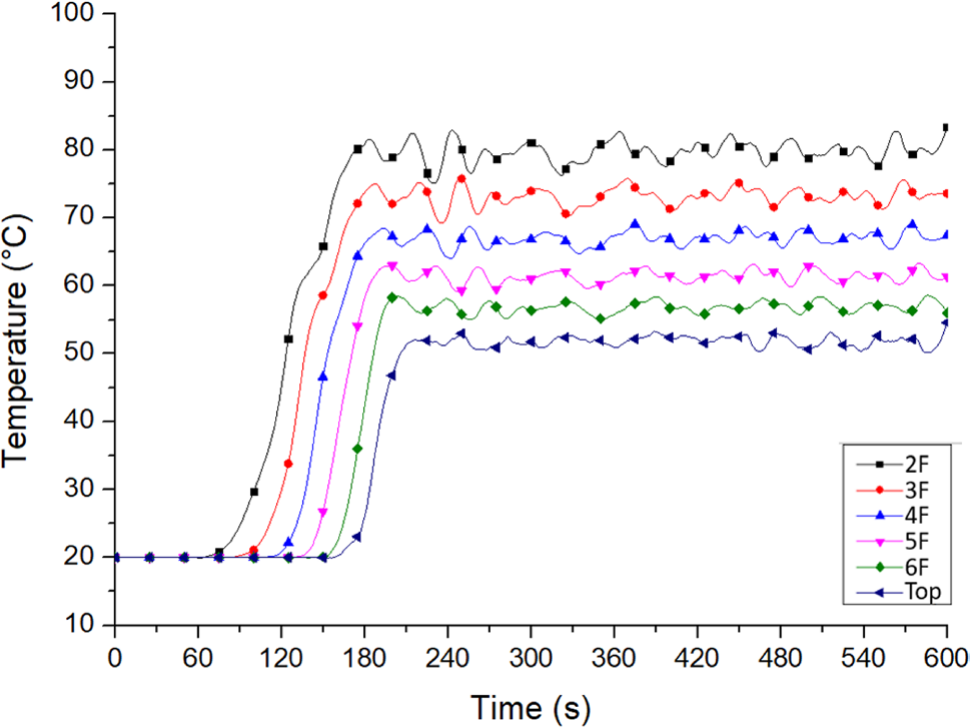
Temperature variation at the resident doors of No. 2 staircase on each floor (for Case To_WAo_B1o).

To further analyze the influence of the stack effect in the building, Case To_WAo_B1o was used to show the hot air distribution, under the assumption that the top doors of Staircase 2 and window Wt were open. Figure 17 shows the temperature distribution from the dwellings on the second floor to the top door. Compared with Fig. 12, it shows that the hot air obviously rose under the condition that the opening at the top of the staircase and window Wt were open. Hence, the sixth floor and the top door temperatures increased from 46 and 40 °C to 56 and 51 °C, respectively.

The simulation results proved that having more staircase openings led to a more obvious phenomenon of hot air rising due to the stack effect described by Klote^41^ and Shi et al.^42^. Therefore, reducing the fire damage could be discussed based on the number of staircase openings or other strategies due to the prevention of the stack effect.

### Improvement strategy 1: change the type of the door in the basement

For the smoke control strategy, the impact of the makeup air on the fire scene is pronounced. As shown in Fig. 3B, all the entrances of the staircases in the basement were equipped with grilled doors, allowing the airflow to pass easily. This is a way to block the inflow of fresh air according to combustion theory^43^.

Due to the hot summers in Taiwan, most dwellings are generally equipped with grilled doors for ventilation and lighting. Unfortunately, the stack effect at a fire site can turn this type of door into an inlet for hot air and smoke. Therefore, the first improvement strategy proposed in this study was to change the type of staircase doors into a closed style to block the hot air inflow. Most doors used in Taiwan are made of metal. The fire will gradually go out when the hot air of the fire cannot rise. This phenomenon confirmed the first term of Eq. (1), that is, the reduction of the F_body_.

Case Tc_Wtc_B1c is the simulation of changing the basement doors to a closed style under existing conditions. In the model, the upper opening vent and window Wt of Staircase 2 were closed, similar to the present situation. Meanwhile, the grilled doors of other staircases in the basement were not changed for convenient comparison. The settings for all windows were the same as those in Case Tc_Wtc_B1o.

**Figure 18:**
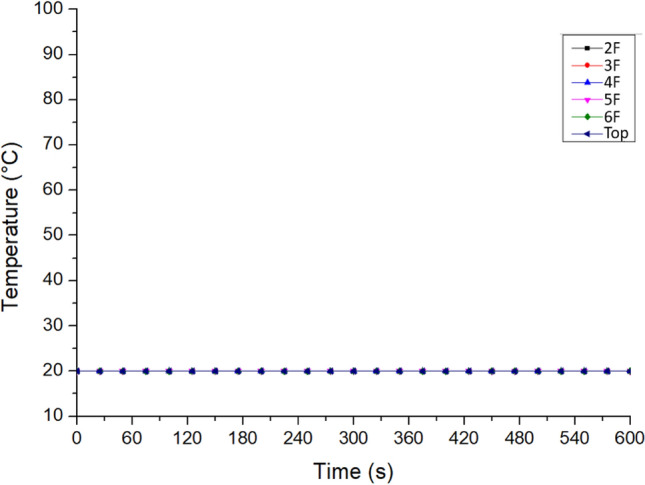
Temperature variation at the resident doors of No. 2 staircase on each floor (for Case Tc_Wtc_B1c).

Figure 18 shows the temperature distribution at all residents’ doors on each floor near Staircase 2. The graph shows a straight line of 20 °C. The doors in the basement blocked the hot air and smoke, preventing the stack effect. If the hot air failed to enter the staircases, the temperature at all residents’ doors on all floors would not rise and would be maintained at 20 °C. In other words, when the fire broke out, the residents would not have been exposed to hot air and smoke if the basement doors of the building were fully enclosed rather than grilled. The simulation results proved that it was an improvement strategy worthy of reference.

### Improvement strategy 2: closing the windows of the stairs on all floors at night

Windows are mandatory requirements imposed by building codes. The fire scene investigation showed that most of the windows in the staircases were usually open. Fires at night have a higher risk of harm because most people are sleeping. This study, considering the reduction of the stack effect, analyzed the hot air distribution when the windows on all floors of Staircase 2 were closed. However, closing windows in such residential buildings may have adverse effects—for instance, on air quality and power consumption. Thus, residents may consider closing the windows of the stairs at night to improve fire safety. Besides, they do not necessarily need to open the windows of the stairs at night.

**Figure 19:**
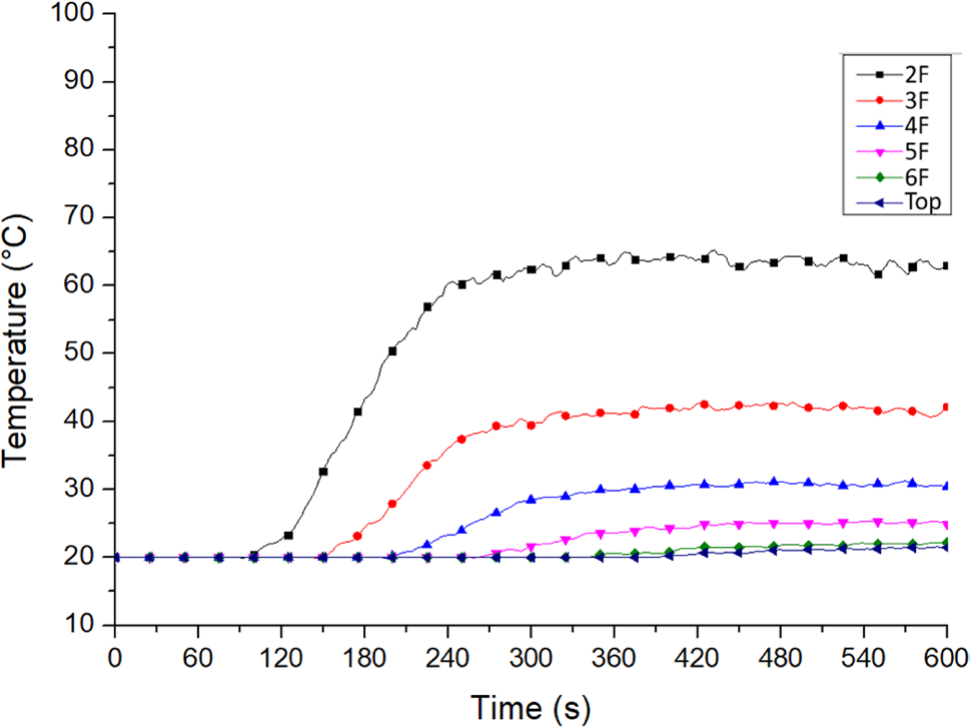
Temperature variation at the resident doors of No. 2 staircase on each floor (for Case Tc_WAc_B1o).

Figure 19 shows the simulation of the temperature variation at all residents’ doors on each floor near Staircase 2. According to the results shown in Table 6 and Fig. 19, the temperature on other floors was lower than 60 °C. The lower temperatures indicated that closing the windows at night in the staircases could effectively restrain the stack effect. However, this finding exempts the 60 °C temperature on the second floor, which is higher than the tolerable temperature of the human body.

When the temperature on the lower floors increased, the staircases became unsuitable for downward evacuation. The rise in temperature in the stairwell can be divided into lower and upper regions, depending on the location and attenuation effect of the upper opening^44^. The upward flow of hot air could be restrained by closing the staircase windows, thereby ensuring the safety of the residents on the upper floors. This phenomenon confirmed the first term of Eq. (1), that is, the reduction of the F_body_. This improvement strategy is also worthy of reference.

**Figure 20:**
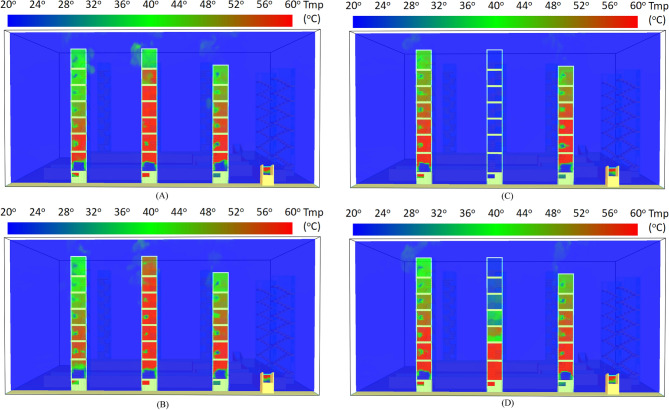
Temperature diffusion inside the 1st, 2nd and 3rd stairs of buildings for each case at 600 s (**A**) Case Tc_Wtc_B1o. (**B**) Case To_WAo_B1o. (**C**) Case Tc_Wtc_B1c. (**D**) Case Tc_WAc_B1o.

Figure 20 shows the temperature distribution at 600 s of Staircase 1 to Staircase 3 in the four case simulations of this study. Figure 20A shows Case Tc_Wtc_B1o and indicates a noticeable stack effect. On the other hand, Fig. 20B shows the simulation results of Case To_WAo_B1o, indicating that more openings at the top would lead to a greater stack effect and greater harm to the residents. Figure 20C shows Case Tc_Wtc_B1c, the first improvement strategy proposed in this study, which was to change the door type in the basement. The results showed that there was no hot air distribution in Staircase 2. This proved that the hot flow from the basement fire could not or did not easily rise up in Staircase 2.

Further, because the doors in the other staircases in the basement were not changed, the situation was more serious. Figure 20D shows Case Tc_WAc_B1o, the second improvement strategy proposed in this study, which was to reduce the number of windows in the staircases to reduce hazards. The results showed that the stack effect in Staircases 2 was greatly diminished and the upward flow of hot air reduced.

## Conclusion

In Taiwan, dwelling fires have accounted for more than 75% of fire accidents in recent years. This study analyzed a dwelling fire that occurred in Tainan City at 00:04 on March 17, 2019. The fire officials regarded it as a major fire, compelling them to dispatch 26 fire engines and 65 firefighters to the scene. A large amount of data was collected during the site investigation. Tainan City is the earliest developed city in Taiwan and holds a history of 350 years. While many buildings are old, the installed fire equipment often only meets the minimum safety criteria of the current fire regulations. In this study, the fire scene was successfully reconstructed, and the results were verified with the actual situation one by one, proving that the simulation was consistent with the actual situation. It was also found that the makeup air phenomenon at the fire site was evident.

The results showed that the stack effect was apparent in the interconnected staircases to the basement. Thus, the residents faced a harsh fire environment four minutes after the fire began. Therefore, two improvement strategies according to the control volume theory of fluid mechanics were proposed in this study and proved to be effective. The first strategy was to change the type of door used in the basement. The fire will gradually go out when the hot air of the fire cannot rise. The results showed that the smoke was effectively prevented from entering the staircases in the building after changing the original grilled doors used for energy conservation to closed flat doors. The second strategy was to close the windows of the stairs on all floors at night. The results showed that it could effectively restrain the stack effect.

The improvement strategies proposed in this paper are immediately feasible and inexpensive. Moreover, the results of this study could provide a reference for other old dwellings, helpful for ensuring the fire safety of residents.

## Supplementary Information

Supplementary Information.
