# Supplementary material for: Stack effects in tall building fires: a case study of Taiwan old apartment fire
Source: Sci Rep. 2022 May 27;12:8963. doi: 10.1038/s41598-022-13118-z (PMC9142533; doi:10.1038/s41598-022-13118-z)
Supplement: Supplementary file 1 — Supplementary Information. [file 41598_2022_13118_MOESM1_ESM.docx]

**Appendix**

&REAC ID='HEPTANE',

FUEL='REAC_FUEL',

FORMULA='C7H16',

CO_YIELD=6.0E-3,

SOOT_YIELD=0.015/

&SURF ID='Burner',

COLOR='RED',

HRRPUA=653.85,

TAU_Q=-169.21/
